# Integrated analysis of gut and oral microbiome in men who have sex with men with HIV Infection

**DOI:** 10.1128/spectrum.01064-23

**Published:** 2023-10-18

**Authors:** Shuang Li, Bin Su, Hao Wu, Qiushui He, Tong Zhang

**Affiliations:** 1 Beijing Key Laboratory for HIV/AIDS Research, Clinical and Research Center for Infectious Diseases, Beijing Youan Hospital, Capital Medical University, Beijing, China; 2 Institute of Biomedicine, Research Center for Infections and Immunity, University of Turku, Turku, Finland; 3 Department of Medical Microbiology, Capital Medical University, Beijing, China; American Type Culture Collection, Manassas, Virginia, USA

**Keywords:** HIV, gut microbiome, oral microbiome, 16S rRNA sequencing, antiretroviral therapy

## Abstract

Human immunodeficiency virus (HIV) infection leads to severe deficiency in host immunity by depletion of CD4^+^ T-cells, resulting in an imbalance between the human microbiome and host immune response. Alterations in both gut and oral microbiomes have been observed in people living with HIV (PLWH). However, the impact of an altered gut and oral microbiome on HIV disease progression and the relationship between them in PLWH have not been explored. In this longitudinal study, we enrolled acute HIV-infected men who have sex with men (MSM) (*n* = 15), chronic HIV-infected MSM (*n* = 15), and HIV-uninfected MSM controls (*n* = 15). We collected anal and throat swab samples at recruitment (W0) from all participants and at 12 wk after antiretroviral therapy (ART) (W12) from the patients and performed 16S rRNA gene sequencing of genomic DNAs extracted from these swabs. In HIV-infected individuals with CD4 T-cell counts < 350 cells/µL, the increase in abundance of *Streptococcus* in the oral microbiome was inversely correlated with that of *Streptococcus* in the gut microbiome (*r* = −0.490, *P* = 0.039). In addition, the lower CD4^+^ T-cell counts (<200 cells/µL) were associated with the higher abundance of *Escherichia–Shigella* and the lower abundance of *Methylobacterium–Methylorubrum* of the gut microbiome in HIV-infected individuals. We demonstrate the alteration of gut microbiome resulting from HIV infection and ART and the relationship between the gut and oral microbiome in PLWH and controls. These findings highlight the need for a better understanding of the interactions between the oral and gut microbiome and its potential role in HIV disease progression.

**IMPORTANCE:**

Our longitudinal integrated study has shown the marked alterations in the gut and oral microbiome resulting from acute and chronic HIV infection and from antiretroviral therapy. Importantly, the relationship between oral and gut microbiomes in people living with acute and chronic HIV infection and “healthy” controls has also been explored. These findings might contribute to a better understanding of the interactions between the oral and gut microbiomes and its potential role in HIV disease progression.

## INTRODUCTION

Although the life expectancy of HIV-infected individuals has been greatly increased by effective antiretroviral therapy (ART), non-AIDS comorbidities have also increased. Further, the non-AIDS morbidity and mortality in HIV-infected individuals receiving ART remain higher when compared with uninfected HIV individuals (1 – 3). The impact of oral and gut microbiome dysbiosis on HIV disease progression is widely acknowledged, but the relationship between gut and oral microbiomes and their potential mechanism in this process remains unclear.

During the early phase of HIV infection, the number of CD4^+^ T-cells, particularly T helper (Th) 17 cell subset, is greatly decreased in the gastrointestinal (GI) tract, leading to a substantial immunological and structural disruption of GI (4, 5). The translocation of the gut microbiome and microbial products across the damaged mucosal barrier into the systemic circulation promotes HIV-associated immune activation and inflammation, which are associated with HIV disease progression and non-AIDS comorbidities onset (5 – 8). In addition, numerous studies have shown that there is a significant difference in the composition of the oral microbiome between HIV-infected individuals and healthy controls (9, 10). The dysbiosis of the oral microbiome in people living with HIV (PLWH) might be associated with altered salivary composition and function, innate and adaptive immune responses, and altered physiological structure and function of the oral mucosa in HIV infection (11).

In addition to changes in the oral and gut microbiome, oral-to-gut and gut-to-oral microbial transmissions might reshape the microbial ecosystem of oral and gut, which plays an important role in regulating the pathogenesis of disease (12). Recent studies have demonstrated substantial effects of the oral–gut microbiome axis on the pathogenesis of various human diseases, such as inflammatory bowel disease (13 – 15), colorectal cancer (16), liver disease (17 – 19), and rheumatoid arthritis (20). In addition, the oral microbiome can shape the lung microbiome; gut microbiome dysbiosis might impact lung diseases such as asthma and alter immune responses to acute respiratory infections such as influenza (21 – 23). Shenoy et al. reported that the gut microbiome in HIV-infected individuals with pneumonia is associated with altered lung microbiome, peripheral CD4 counts, and macrophage dysfunction (22). Another study has explored the relationship between the oral and gut microbiome in patients with HIV-associated chronic obstructive pulmonary disease (COPD). They found that the oral microbiome was altered in PLWH with COPD, and the change in the oral microbiome might contribute to lung function (10). Cribbs et al. demonstrated that gut microbiome dysbiosis might be associated with HIV-related lung disease through the effects on systemic inflammation, microbial translocation, metabolite production, and antimicrobial production (24). However, studies relating the composition of the gut microbiome to the oral or lung microbiome in PLWH are still lacking. Clearly, longitudinal studies with the aim of investigating the alterations of the gut and oral microbiome and the oral–gut microbial interactions in PLWH are needed.

Our previous study has compared and identified changes in the oral microbiome in acute and chronic HIV-infected men who have sex with men (MSM) prior to and after 12 wk of ART (25). In this present study, we performed 16S rRNA sequencing of anal swabs collected from the same cohorts of patients and controls. We compared the results obtained from both anal and throat swabs and explored the changes in the gut microbiome, as well as the oral–gut microbial interactions in MSM with acute and chronic HIV infection before and after ART and HIV-negative MSM controls.

## MATERIALS AND METHODS

### Study subjects and sample collection

From May to November 2019, we recruited 30 MSM with acute HIV infection (*n* = 15) and chronic HIV infection (*n* = 15) and 15 HIV-uninfected MSM controls from Beijing Youan Hospital, Beijing, China. All MSM with acute and chronic HIV infection enrolled in our study had not initiated ART. At the time of recruitment, throat swabs were collected from acute HIV-infected ART-naive individuals (referred to as Oral_Acute HIV, *n* = 15) and chronic HIV-infected ART-naive individuals (Oral_Chronic HIV, *n* = 15), and anal swabs were obtained from acute HIV-infected ART-naive individuals (Gut_Acute HIV, *n* = 15) and chronic HIV-infected ART-naive individuals (Gut_Chronic HIV, *n* = 15). We also collected throat swabs (Oral_Non-infected, *n* = 15) and anal swabs (Gut_Non-infected, *n* = 15) from HIV-uninfected controls at the time of recruitment. Thereafter, all HIV-infected individuals received ART. At 12 wk of ART, throat swabs were collected from acute HIV-infected individuals (Oral_Acute ART, *n* = 13) and chronic HIV-infected individuals (Oral_Chronic ART, *n* = 15), and anal swabs were obtained from acute HIV-infected individuals (Gut_Acute ART, *n* = 13) and chronic HIV-infected individuals (Gut_Chronic ART, *n* = 15) (two patients were lost to follow-up at 12 wk of ART). In this study, the ART regimen of most HIV-infected individuals is tenofovir disoproxil fumarate (TDF) + lamivudine (3TC)+efavirenz (EFV), three of the HIV-infected patients were treated with TDF + 3TC +dolutegravir (DTG), and two patients were treated with TDF + 3TC +lopinavir/ritonavir (LPV/r).

Acute HIV infection was defined as a positive HIV RNA but a negative or indeterminate HIV antibody result. Chronic HIV infection was diagnosed through various tests, primarily the detection of HIV antibodies or viral RNA/DNA in the blood. In addition, we excluded participants with opportunistic infection and hepatitis B or C viruses. The individuals who had used antibiotics, probiotics, and prebiotics during the previous 4 wk were excluded. Furthermore, we also excluded recreational drug and injection drug users. The participant demographic and clinical characteristics are presented in Table 1.

**TABLE 1:** Demographic and clinical characteristics of study participants^*a*^

Parameters	Acute HIV infection	Chronic HIV infection	Controls
Number of subjects	15	15	15
MSM	15	15	15
Age (years)	28 (19–50)	33 (23–55)	40 (26–55)
CD4^+^ T-cell counts (cells/μL)			
Pre-ART	453 (86–1,324)	410 (27–659)	NA^*b*^
ART	396 (150–577)	498 (93–1,021)	NA
Viral load (copies/mL)	28,028 (3,493–200,728)	10,907 (545–257,327)	NA

^*a*^ Data are presented as *n* or median (range).

^*b*^ NA: Not applicable

This study and other related experiments received approval from the Beijing Youan Hospital Research Ethics Committee ([2018]025), and all study participants have provided written informed consent.

### 16S rRNA gene sequence analysis

The anal and throat swab samples were collected by clinical nurses from PLWH and HIV-uninfected controls and quickly sent to and stored at −20°C in the laboratory for further analysis. We used the QIAamp Fast DNA Stool Mini Kit (50) (QIAGEN, Hilden, Germany) to extract genomic DNAs from anal and throat swabs. The genomic DNAs were amplified by PCR for the 16S rRNA V4–V5 region sequencing. The Illumina TruSeq DNA PCR-Free Library Preparation Kit (Illumina, USA) was used to generate the sequencing libraries, and the Qubit@ 2.0 Fluorometer (Thermo Scientific) and Agilent Bioanalyzer 2100 system were used to assess the quality of the library. After that, the library was sequenced on Illumina NovaSeq platform and generated 250 bp paired-end reads. Based on the unique barcodes, the paired-end reads were assigned to samples and merged as raw tags by using FLASH (V1.2.7). The raw tags were finally obtained. Based on the QIIME (V1.9.1, http://qiime.org/scripts/split_libraries_fastq.html) quality control process, the high-quality clean tags were obtained by quality filtering under specific filtering conditions, and the tags were used to compare with the reference database (Silva database, https://www.arb-silva.de/) to detect chimera sequences by the UCHIME algorithm (UCHIME algorithm, http://www.drive5.com/usearch/manual/uchime_algo.html). After removing the chimeric sequences, we finally obtained the effective tags.

By using Uparse software (Uparse v7.0.1001), the operational taxonomic units (OTUs) were clustered for 16S rRNA gene sequences with 97% similarity (26). The alpha diversity indices (27), including observed species, Chao1, Shannon, Simpson, ACE, good coverage, and PD whole tree, were calculated with QIIME (Version 1.7.0) and displayed with R software (Version 2.15.3). The beta diversity analysis on weighted and unweighted UniFrac was calculated by QIIME software (Version 1.9.1) (28). Principal coordinate analysis (PCoA) was showed by using WGCNA package, stat packages, and ggplot2 package in R software (Version 2.15.3) (28).

### Statistical analysis

The alpha diversity and beta diversity in the gut and oral microbiome among groups were tested by the Wilcoxon rank-sum test. To further evaluate the differences in microbial abundance and diversity between samples, statistical analysis methods were used for the significance test. Linear discriminant analysis (LDA) effect size (LefSe) analysis and *t*-test were used to evaluate the differences in the composition of the gut microbiome in people living with acute HIV infection and chronic HIV infection before or after ART. MetaStat analysis was used to compare the composition of gut and oral microbiomes among different groups. Correlations between two variables were analyzed in non-parametric Spearman’s rank correlation tests, with *r* being the Spearman correlation coefficient. The Mann–Whitney test and Kruskal–Wallis test were used to compare continuous variables. Two sides of *P* < 0.05 were considered to be statistically significant.

## RESULTS

### Study population

The anal and throat swab samples were collected from 15 acute HIV-infected individuals, 15 chronic HIV-infected individuals, and 15 healthy controls. All study participants (≥18 years old) were defined as MSM. Altogether, 146 samples were included in this study.

### Gut microbiome dysbiosis in people living with acute and chronic HIV infection

**Fig 1:**
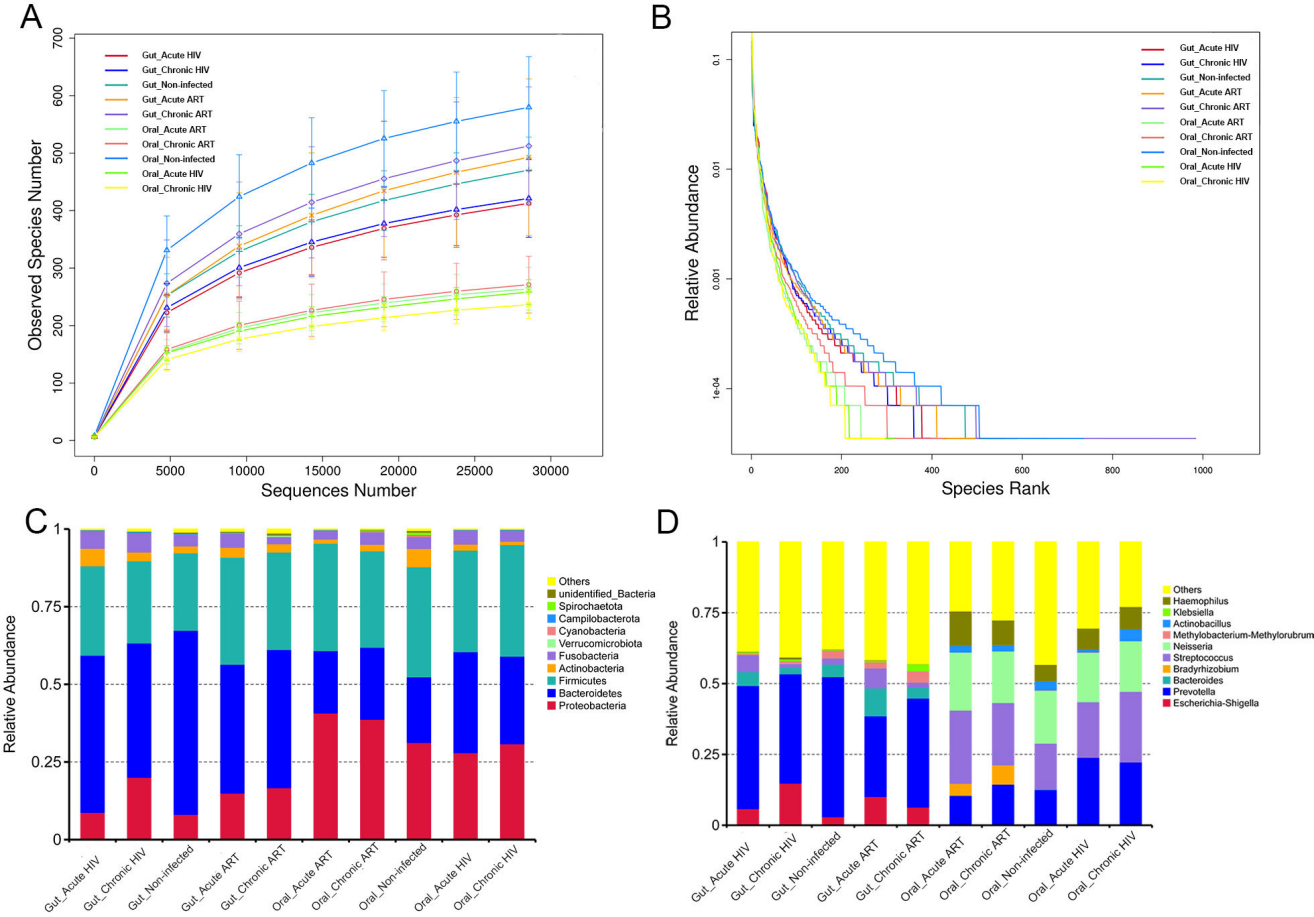
The composition and relative abundance of the oral and gut microbiome in HIV-infected groups and controls. (**A**) Rarefaction curve of the OTUs; (**B**) Rank abundance of the OTUs; (**C**) at the phylum level; (**D**) at the genus level; Oral_Acute HIV: oral microbiome in people living with acute HIV infection at baseline; Oral_Chronic HIV: oral microbiome in people living with chronic HIV infection at baseline; Oral_Non-infected: oral microbiome in HIV-uninfected controls; Oral_Acute ART: oral microbiome in people living with acute HIV infection after 12 wk of ART; Oral_Chronic ART: oral microbiome in people living with chronic HIV infection after 12 wk of ART.

Sequencing of anal swab and throat swab samples resulted in an average of 55,180 and 58,819 effective tags for subsequent analysis. The rarefaction curves indirectly reflected the abundance of the gut and oral microbiome and showed a great sequencing depth of our study (Fig. 1A). The rank abundance showed an even distribution of species of the samples in our study (Fig. 1B). Composition of the gut and oral microbiome in controls and people living with acute and chronic HIV infection before and after ART were analyzed in our study. Similar to oral microbiome, the most abundantly detected bacterial phyla in the gut microbiome were Bacteroidetes, Firmicutes, Proteobacteria, Fusobacteria, and Actinobacteria (Fig. 1C; Table S1). These five dominant bacterial phyla accounted for more than 98% of the total gut microbiome. However, the Proteobacteria accounted for the highest proportion of all members of the oral microbiome, while the Bacteroidetes accounted for the highest proportion of the gut microbiome. The following dominant genera were observed in the gut microbiome: *Prevotella*, *Bacteroides*, *Escherichia–Shigella*, *Methylobacterium–Methylorubrum*, and *Streptococcus* (Table S2). However, *Streptococcus*, *Neisseria*, *Prevotella*, *Haemophilus,* and *Actinobacillus* dominate the oral microbiome. In addition, the abundance of *Bradyrhizobium* in the oral microbiome was increased in both acute and chronic HIV infection groups following 12 wk of ART, whereas no changes in the abundance of *Bradyrhizobium* were found in the gut microbiome (Fig. 1D; Table S2).

**Fig 2:**
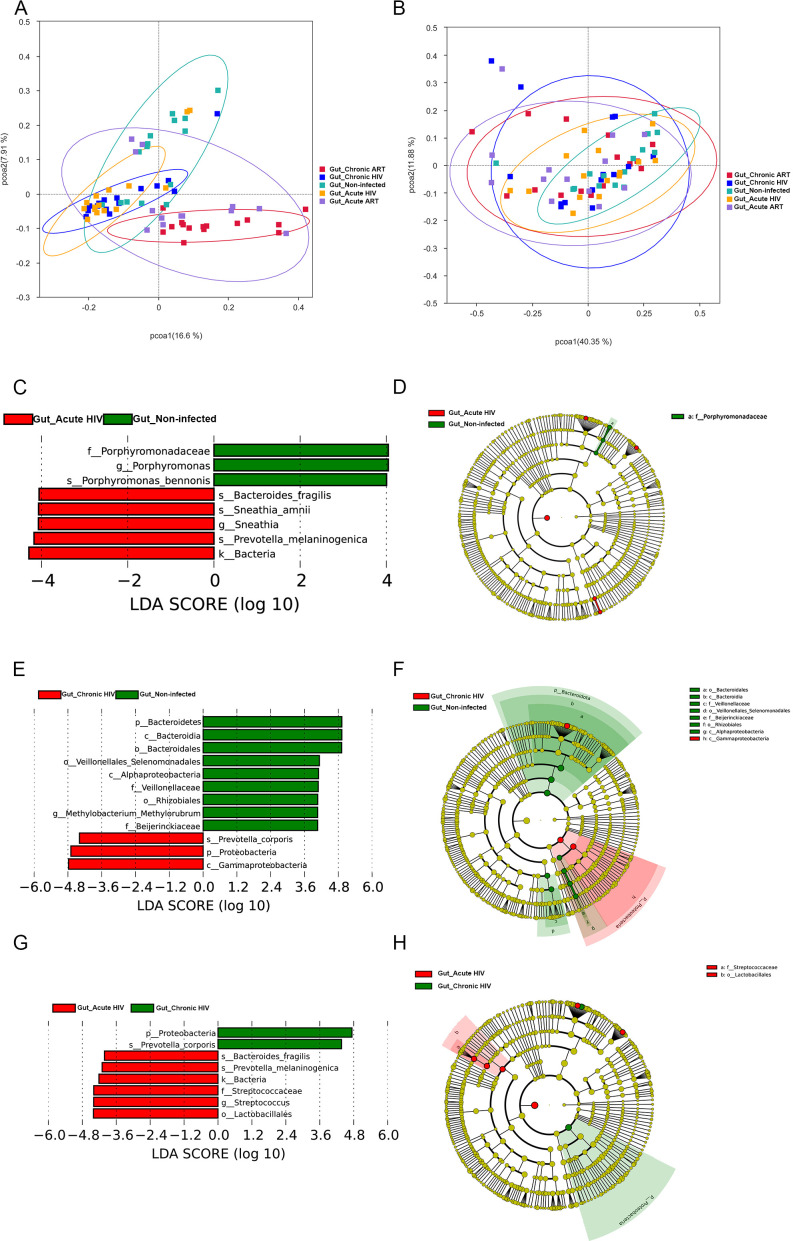
The diversity of the gut microbiome in HIV-infected groups and controls. (**A**) Beta diversity represented by principal coordinate analysis (PCoA) of unweighted UniFrac distances; (**B**) Beta diversity represented by PCoA of weighted UniFrac distances. Linear discriminant analysis (LDA) effect size (LefSe) at the genus level. In C and D, LDA scores for the significant taxa in the Gut_Non-infected group are represented on the positive scale (green), and LDA-negative scores represent enriched taxa in the Gut_Acute HIV group (red); in E and F, LDA scores for the significant taxa in the Gut_Non-infected group are represented on the positive scale (green), and LDA-negative scores represent enriched taxa in the Gut_Chronic HIV group (red); and in G and H, LDA scores for the significant taxa in the Gut_Chronic HIV group are represented on the positive scale (green), and LDA-negative scores represent enriched taxa in the Gut_Acute HIV group (red). Gut_Acute HIV: gut microbiome in people living with acute HIV infection at baseline; Gut_Chronic HIV: gut microbiome in people living with chronic HIV infection at baseline; Gut_Non-infected: gut microbiome in HIV-uninfected controls; Gut_Acute ART: gut microbiome in people living with acute HIV infection after 12 wk of ART; Gut_Chronic ART: gut microbiome in people living with chronic HIV infection after 12 wk of ART.

We next used the alpha diversity index to characterize microbial diversity, evenness of species distribution, and sequencing depth among different groups of samples (Tables S3 and S4). The observed species identified in Gut_Non-infected and Oral_Non-infected groups were 470 and 579, respectively. The corresponding numbers identified in the gut and oral microbiome in acute or chronic HIV-infected individuals before ART were significantly lower than controls (all *P* < 0.05) (Tables S3 and S4). The observed species of the gut microbiome were increased in people living with acute and chronic HIV-infected individuals after 12 wk of ART, and no significant difference was observed between acute and chronic HIV-treated groups and controls (Tables S3 and S4). However, the observed species were still significantly decreased in the Oral_Acute ART and Oral_Chronic ART groups when compared with that of the Oral_Non-infected group (*P* < 0.001) (Tables S3 and S4). The Chao1 index of the Gut_Acute HIV and Gut_Chronic HIV groups was also significantly lower than that of the Gut_Non-infected group (all *P* < 0.05), whereas the difference became not significant after 12 wk of ART (Tables S3 and S4). In addition, we used the PCoA for beta diversity analysis to identify the differences in microbial community composition. A conspicuous separation between the acute and chronic HIV infection groups and the control group was observed (Fig. 2A and B); the beta diversity of the gut microbiome in people living with acute and chronic HIV infection was significantly different from that in HIV-uninfected controls at W0, and the significant difference remained at W12 (all *P* < 0.05) (Fig. 2B; Table S4). However, there was no significant difference in beta diversity of the gut microbiome when comparing acute HIV-infected individuals and chronic HIV-infected individuals regardless of ART (Fig. 2B; Table S4).

Differences in the composition of the gut microbiome between people living with acute and chronic HIV infection and controls were also analyzed in our study. In the acute HIV-infected individuals, *Bacteroides fragilis*, *Sneathia*, and *Prevotella melaninogenica* were enriched in the Gut_Acute HIV group; *Porphyromonas*, *Parasutterella*, *Ruminococcus*, and *Subdoligranulum* were found in greater abundance in the Gut_Non-infected group (Fig. 2C and D; Fig. S1A). At the phylum level, we observed that *Proteobacteria* was enriched in the Gut_Chronic HIV group; *Bacteroidetes* was enriched in the Gut_Non-infected group. In addition, *Prevotella corporis* was enriched in the Gut_Chronic HIV group, whereas *Methylobacterium–Methylorubrum* was enriched in the Gut_Non-infected group (Fig. 2E and F). We also compared the composition of the gut microbiome in acute and chronic HIV-infected individuals. LEfSe analyses showed that *Bacteroides fragilis*, *Prevotella melaninogenica,* and *Streptococcus* were enriched in the Gut_Acute HIV group, whereas *Prevotella corporis* and *Proteobacteria* were enriched in the Gut_Chronic HIV group (Fig. 2G and H). At the genus level, *Catenibacterium*, *Ruminococcus,* and *Bilophila* in the Gut_Chronic HIV group were significantly increased compared with the Gut_Acute HIV group (Fig. S1B).

### Effects of ART on the gut microbiome in people living with acute and chronic HIV infection

**Fig 3:**
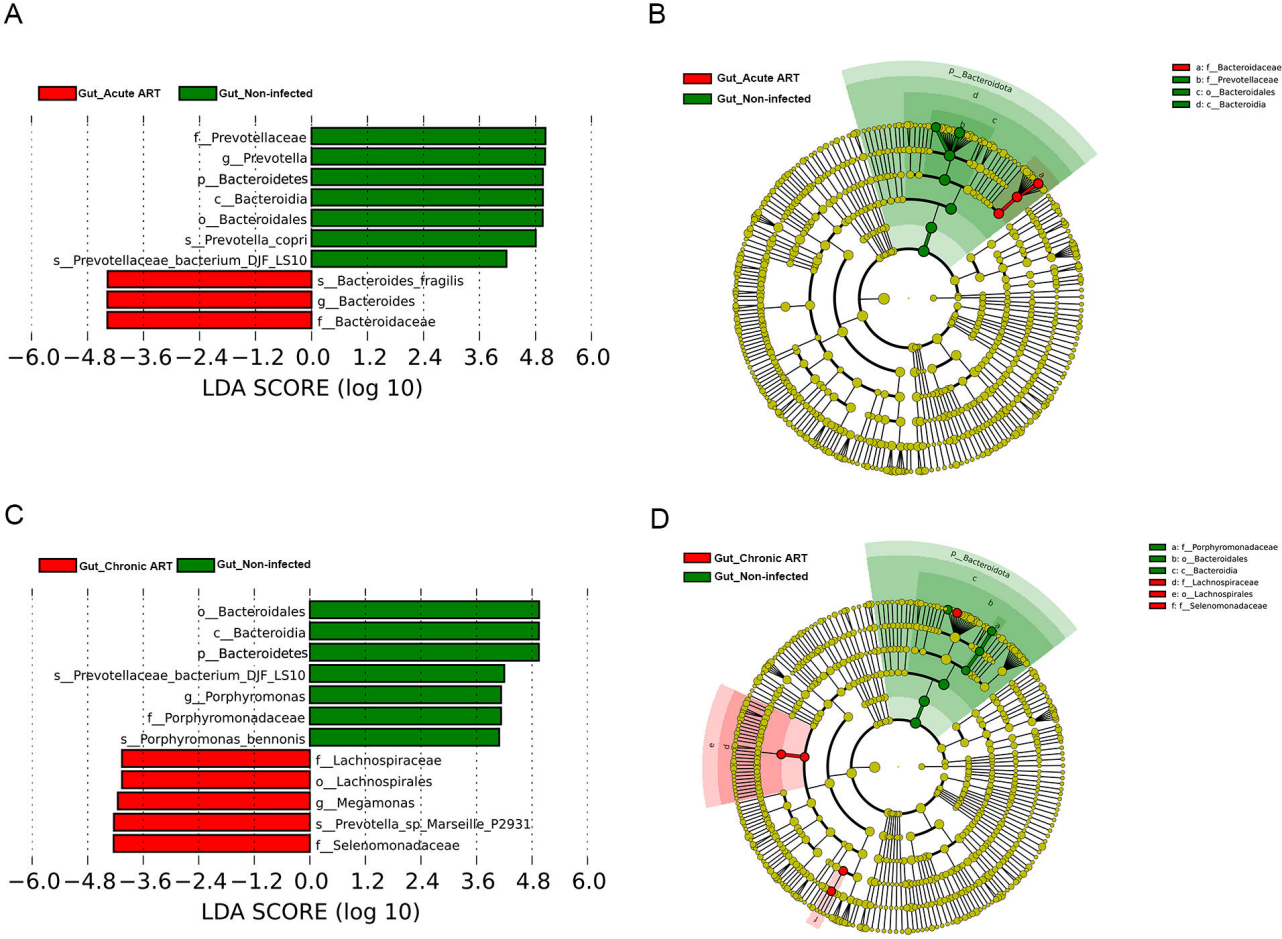
Linear discriminant analysis (LDA) effect size (LefSe) at the genus level. In A and B, LDA scores for the significant taxa in the Gut_Non-infected group are represented on the positive scale (green), and LDA-negative scores represent enriched taxa in the Gut_Acute ART group (red); in C and D, LDA scores for the significant taxa in the Gut_Non-infected group are represented on the positive scale (green), and LDA-negative scores represent enriched taxa in the Gut_Chronic ART group (red). Gut_Acute ART: gut microbiome in people living with acute HIV infection after 12 wk of ART; Gut_Chronic ART: gut microbiome in people living with chronic HIV infection after 12 wk of ART; Gut_Non-infected: gut microbiome in HIV-uninfected controls.

In our study, all subjects with acute and chronic HIV infection received ART following the collection of swab samples at baseline. We collected the second swabs from PLWH after 12 wk of ART. LEfSe analyses demonstrated that *Bacteroides* was enriched in the Gut_Acute ART group, whereas *Prevotella* was enriched in the Gut_Non-infected group (Fig. 3A and B; Fig. S1C). In the chronic HIV-infected individuals after 12 wk of ART, the *Megamonas* and *Phascolarctobacterium* were enriched in the Gut_Chronic ART group, whereas *Porphyromonas* and *Haemophilus* were enriched in the Gut_Non-infected group (Fig. 3C and D; Fig. S1D).

**Fig 4:**
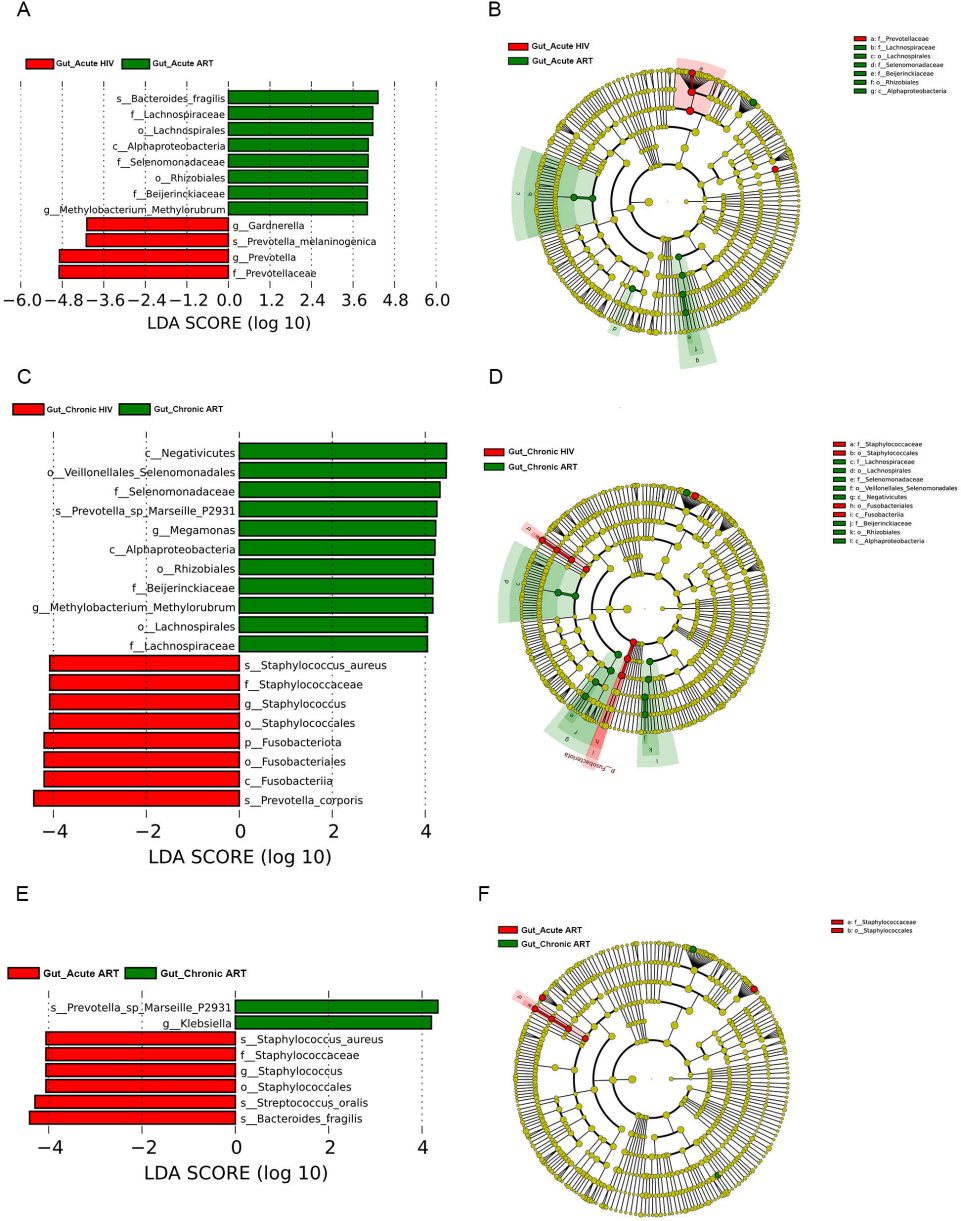
Linear discriminant analysis (LDA) effect size (LefSe) at the genus level. In A and B, LDA scores for the significant taxa in the Gut_Acute ART group are represented on the positive scale (green), and LDA-negative scores represent enriched taxa in the Gut_Acute HIV group (red); in C and D, LDA scores for the significant taxa in the Gut_Chronic ART group are represented on the positive scale (green), and LDA-negative scores represent enriched taxa in the Gut_Chronic HIV group (red); in E and F, LDA scores for the significant taxa in the Gut_Chronic ART group are represented on the positive scale (green), and LDA-negative scores represent enriched taxa in the Gut_Acute ART group (red). Gut_Acute HIV: gut microbiome in people living with acute HIV infection at baseline; Gut_Chronic HIV: gut microbiome in people living with chronic HIV infection at baseline; Gut_Non-infected: gut microbiome in HIV-uninfected controls; Gut_Acute ART: gut microbiome in people living with acute HIV infection after 12 wk of ART; Gut_Chronic ART: gut microbiome in people living with chronic HIV infection after 12 wk of ART.

To further explore the effect of ART on the gut microbiome, we compared the differences in the composition of the gut microbiome in HIV-infected individuals before and after ART. LEfSe analyses showed that *Gardnerella*, *Prevotella,* and *Prevotella melaninogenica* were enriched in the Gut_Acute HIV group, while *Lachnospiriaceae*, *Methylobacterium–Methylorubrum,* and *Bacteroides fragilis* were enriched in the Gut_Acute ART group (Fig. 4A and B; Fig. S1E). In the chronic HIV-infected individuals, *Staphylococcus*, *Prevotella corporis,* and *Gardnerella* were enriched in the Gut_Chronic HIV group, whereas the relative abundance of *Megamonas*, *Methylobacterium–Methylorubrum*, *Lachnospira,* and *Roseburia* in the Gut_Chronic ART group was significantly higher (Fig. 4C and D; Fig. S1F).

In addition, we also compared the composition of the gut microbiome in people living with acute HIV infection and chronic HIV infection after 12 wk of ART. *Staphylococcus*, *Bacteroides fragilis,* and *Streptococcus oralis* were enriched in the Gut_Acute ART group, whereas *Klebsiella* and *Phascolarctobacterium* were enriched in the Gut_Chronic ART group (Fig. 4E and F; Fig. S1G).

### Comparison of the composition of gut and oral microbiomes in HIV infections and healthy controls

**Fig 5:**
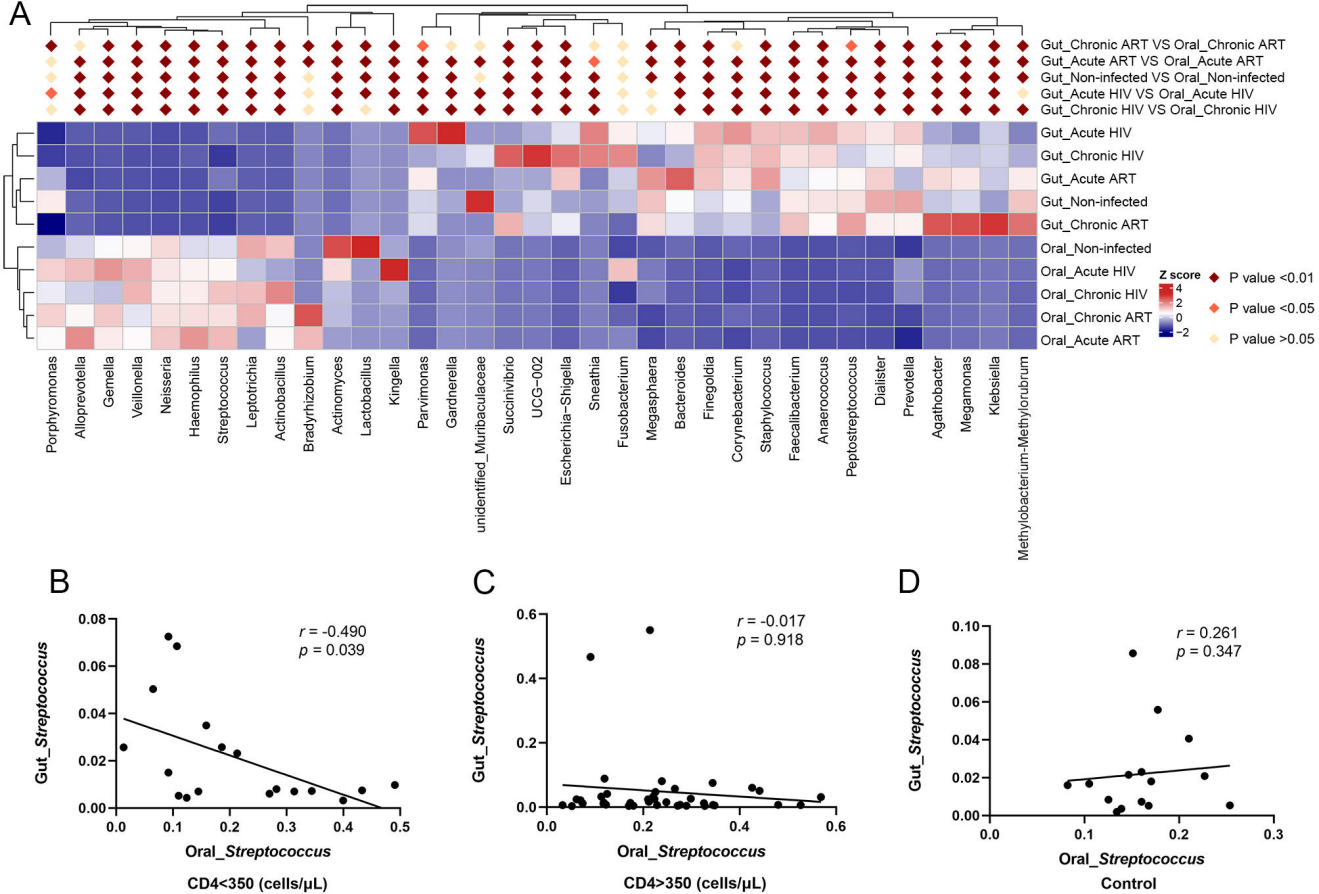
Comparison of the composition of gut and oral microbiomes in HIV infections and healthy controls. (**A**) Comparisons of the relative abundance between oral and gut microbiomes using MetaStat analysis at the genus level. (**B**) Correlations between the abundance of *Streptococcus* in the oral microbiome and the abundance of *Streptococcus* in the gut microbiome in HIV-infected subjects with CD4 <350 cells/µL. (**C**) Correlations between the abundance of *Streptococcus* in the oral microbiome and the abundance of *Streptococcus* in the gut microbiome in HIV-infected subjects with CD4 >350 cells/µL. (**D**) Correlations between the abundance of *Streptococcus* in the oral microbiome and the abundance of *Streptococcus* in the gut microbiome in HIV-uninfected controls. MetaStat analysis was used to compare the composition of gut and oral microbiomes among different groups. Correlations between two variables were analyzed in non-parametric Spearman’s rank correlation tests, with *r* being the Spearman correlation coefficient, and *P* < 0.05 was considered to be statistically significant. Oral_Acute HIV: oral microbiome in people living with acute HIV infection at baseline; Oral_Chronic HIV: oral microbiome in people living with chronic HIV infection at baseline; Oral_Non-infected: oral microbiome in HIV-uninfected controls; Oral_Acute ART: oral microbiome in people living with acute HIV infection after 12 wk of ART; Oral_Chronic ART: oral microbiome in people living with chronic HIV infection after 12 wk of ART. Gut_Acute HIV: gut microbiome in people living with acute HIV infection at baseline; Gut_Chronic HIV: gut microbiome in people living with chronic HIV infection at baseline; Gut_Non-infected: gut microbiome in HIV-uninfected controls; Gut_Acute ART: gut microbiome in people living with acute HIV infection after 12 wk of ART; Gut_Chronic ART: gut microbiome in people living with chronic HIV infection after 12 wk of ART.

To compare the composition of gut and oral microbiomes among different groups of samples, we used MetaStat analysis to investigate bacterial genera with significant differences among the top genera in abundance in the gut and oral microbiomes. In the pre- and post-ART acute and chronic HIV-infected groups and HIV-uninfected controls, the abundances of *Klebsiella*, *Megamonas*, *Agathobacter*, *Prevotella*, *Dialister*, *Peptostreptococcus*, *Anaerococcus*, *Faecalibacterium*, *Staphylococcus*, *Finegoldia*, *Bacteroides*, *Escherichia–Shigella*, *UCG-002*, *Succinivibrio*, *Parvimonas*, *Kingella*, *Actinomyces*, *Actinobacillus*, *Leptotrichia*, *Streptococcus*, *Haemophilus*, *Neisseria*, *Veillonella*, and *Gemella* in the gut microbiome were significantly different from those in the oral microbiome. However, there was no significant difference in the abundance of *Fusobacterium* between the gut microbiome and the oral microbiome among these groups (Fig. 5A). The abundances of *Methylobacterium–Methylorubrum*, *Megasphaera,* and *Lactobacillus* were significantly different between the Gut_Non-infected and Oral_Non-infected groups. However, in both acute and chronic HIV-infected individuals prior to ART, no significant difference was observed in the abundance of *Megasphaera* between gut and oral microbiomes. In addition, there was no significant difference in the abundance of *Methylobacterium–Methylorubrum* between the Gut_Acute HIV and Oral_Acute HIV groups and the abundance of *Lactobacillus* between the Gut_Chronic HIV and Oral_Chronic HIV groups. After 12 wk of ART, these differences in the abundances of *Methylobacterium–Methylorubrum*, *Megasphaera, and Lactobacillus* between gut and oral microbiomes became significant in acute and chronic HIV-infected individuals (Fig. 5A).

In addition, no significant difference was observed in the abundances of *Porphyromonas* and *Bradyrhizobium* between the gut microbiome and the oral microbiome in the healthy controls. However, there was a significant difference in the abundance of *Porphyromonas* between the Gut_Chronic ART and Oral_Chronic ART groups and the abundance of *Bradyrhizobium* between gut and oral microbiomes in both acute and chronic HIV-infected individuals after 12 wk of ART. In contrast, there was a significant difference in the abundances of *Corynebacterium*, *Sneathia*, *Gardnerella,* and *Alloprevotella* between the gut microbiome and the oral microbiome in the healthy controls and acute and chronic HIV-infected individuals prior to ART. However, there was no significant difference in the abundances of *Corynebacterium*, *Sneathia*, *Gardnerella,* and *Alloprevotella* between the Gut_Chronic ART and Oral_Chronic ART groups (Fig. 5A).

To further analyze relations between gut and oral microbiomes in HIV-infected groups and HIV-uninfected controls, the Spearman rank correlation test was performed among top genera in abundance in gut and oral microbiomes. In the HIV-infected subjects with CD4 <350 cells/µL, the correlation analysis revealed that the increase in the abundance of *Streptococcus* in the oral microbiome was inversely associated with the abundance of *Streptococcus* in the gut microbiome (*r* = −0.490, *P* = 0.039; Fig. 5B). However, this relationship was not seen between *Streptococcus* in the oral microbiome and *Streptococcus* in the gut microbiome in the HIV-infected subjects with CD4 >350 cells/µL (*r* = −0.017, *P* = 0.918; Fig. 5C) and the HIV-uninfected controls (*r* = 0.261, *P* = 0.347; Fig. 5D).

### Association of the gut microbiome with CD4^+^ T-cell count

**Fig 6:**
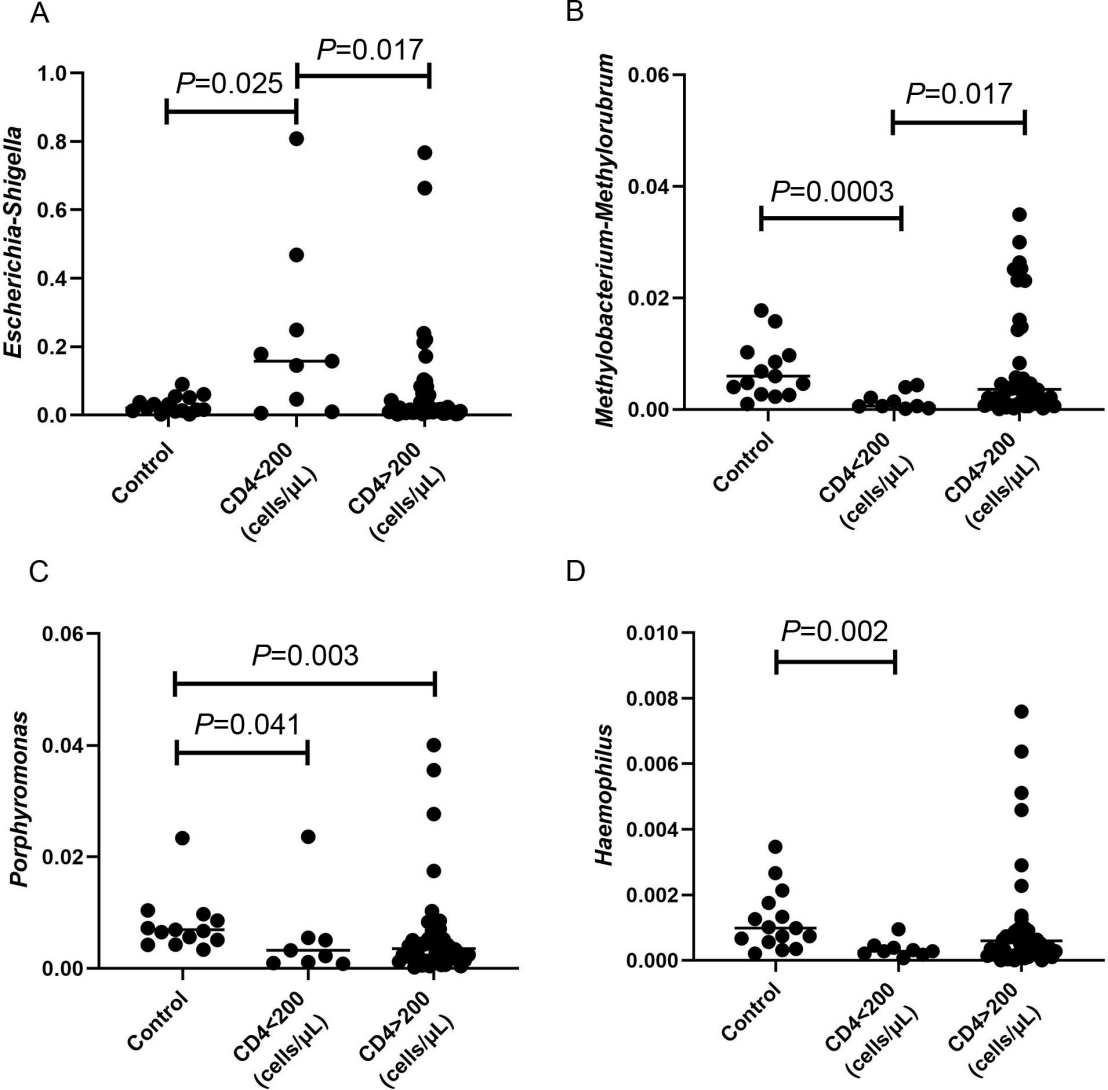
Comparison of relative abundances of (**A**) *Escherichia–Shigella*, (**B**) *Methylobacterium–Methylorubrum*, (**C**) *Porphyromonas,* and (**D**) *Haemophilus* collected from subjects whose CD4^+^ T-cells were defined <200 and CD4 >200 cells/µL and HIV-uninfected MSM controls. The Mann–Whitney test and Kruskal–Wallis test were used to compare continuous variables, and *P* < 0.05 was considered to be statistically significant.

We investigated the relationship between the alteration of the gut microbiome and CD4^+^ T-cell counts in our study. We found that the abundances of *Methylobacterium–Methylorubrum*, *Porphyromonas,* and *Haemophilus* collected from samples of HIV-infected subjects with CD4 <200 cells/µL were significantly lower than those in HIV-uninfected controls (all *P* < 0.05). However, the abundance of *Escherichia–Shigella* collected from samples of HIV-infected subjects with CD4 <200 cells/µL was significantly higher than those in HIV-uninfected controls (*P* < 0.05). In addition, the lower CD4^+^ T-cell counts (<200 cells/µL) were associated with the higher relative abundance of *Escherichia–Shigella* and the lower relative abundance of *Methylobacterium–Methylorubrum* (all *P* < 0.05) (Fig. 6). In addition, no significant correlation was found between the viral load and gut microbiomes. Moreover, there are no different correlations when comparing the gut microbiomes of individuals with “high” loads (>10,000 copies/mL) and with “low” loads (<10,000 copies/mL) in our study. Moreover, we used the PCoA for beta diversity analysis in HIV-infected subjects with CD4 <200 cells/µL and CD4 >200 cells/µL groups and controls to identify the differences in microbial community composition. The CD4^+^ T-cell count of HIV-infected individuals can affect the diversity of the gut microbiome (Fig. S2).

### Microbiome metabolic pathways in gut and oral microbiome in HIV infections and healthy controls

**Fig 7:**
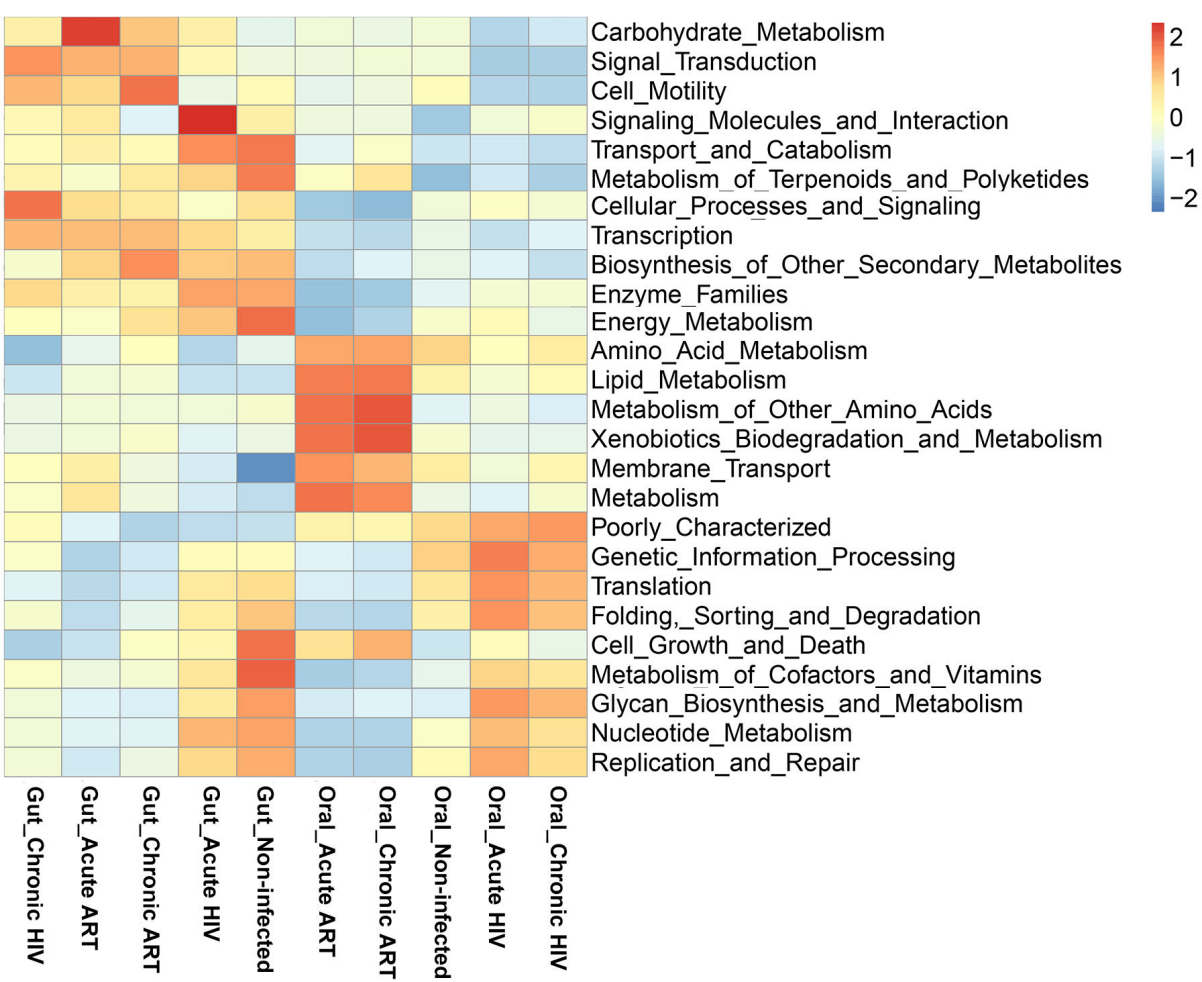
Heatmap and hierarchical clustering of pathways. Oral_Acute HIV: oral microbiome in people living with acute HIV infection at baseline; Oral_Chronic HIV: oral microbiome in people living with chronic HIV infection at baseline; Oral_Non-infected: oral microbiome in HIV-uninfected controls; Oral_Acute ART: oral microbiome in people living with acute HIV infection after 12 wk of ART; Oral_Chronic ART: oral microbiome in people living with chronic HIV infection after 12 wk of ART. Gut_Acute HIV: gut microbiome in people living with acute HIV infection at baseline; Gut_Chronic HIV: gut microbiome in people living with chronic HIV infection at baseline; Gut_Non-infected: gut microbiome in HIV-uninfected controls; Gut_Acute ART: gut microbiome in people living with acute HIV infection after 12 wk of ART; Gut_Chronic ART: gut microbiome in people living with chronic HIV infection after 12 wk of ART.

Based on the functional annotation**,** the PICRUSt metagenome prediction was used to estimate the functional role of the gut microbiome in HIV-infected individuals and healthy controls. Compared to the Gut_Non-infected group, the signaling molecules and interaction-related pathway were increased in the Gut_Acute HIV group (*P* < 0.05); pathways related to membrane transport, signal transduction, and transcription were significantly increased. However, energy metabolism, metabolism of cofactors and vitamins, metabolism of terpenoids and polyketides, cell growth and death, and transport and catabolism were significantly decreased in the Gut_Chronic HIV group (all *P* values < 0.05) (Fig. 7; Fig. S3A and 3B). In addition, the pathway related to cell motility was significantly increased; transport and catabolism and signaling molecules and interaction-related pathways were significantly decreased in the Gut_Chronic HIV group when compared with the Gut_Acute HIV group (Fig. 7; Fig. S3C).

After 12 wk of ART, pathways involved in membrane transport and carbohydrate metabolism were significantly increased (*P* < 0.05). Metabolism-related pathways including energy metabolism, metabolism of cofactors and vitamins, nucleotide metabolism, glycan biosynthesis and metabolism, and enzyme families and genetic information processing-related pathways including translation, folding, sorting, and degradation were significantly decreased in both Gut_Acute ART and Gut_Chronic ART groups when compared with the Gut_Non-infected group (all *P* values < 0.05) (Fig. 7; Fig. S3D and E). Compared with the Gut_Acute HIV group, pathways related to replication and repair, translation, nucleotide metabolism, folding, sorting and degradation, and enzyme families were significantly decreased, and the pathway related to cell motility was significantly increased in the Gut_Acute ART group (*P* < 0.05). In the chronic HIV-infected individuals, amino acid metabolism and biosynthesis of other secondary metabolites–related pathways in the Gut_Chronic ART group were significantly increased when compared with the Gut_Chronic HIV group (*P* < 0.05) (Fig. 7; Fig. S3F and G).

The pathways between gut and oral microbiome in HIV-uninfected controls and HIV infections were further analyzed. In controls, we found that metabolism-related pathways including energy metabolism, metabolism of cofactors and vitamins, nucleotide metabolism, glycan biosynthesis and metabolism, and enzyme families and genetic information processing–related pathways including transcription, cell growth and death, transport, and catabolism were significantly increased in the gut microbiome when compared with the oral microbiome. However, HIV infection can affect these metabolic pathways in oral and gut microbiomes, especially in patients with chronic HIV infection, and ART can only provide partial recovery (Fig. 7; Fig. S4).

## DISCUSSION

In this study, we found that the gut microbiome shared similar common bacterial phyla to those in the oral microbiome, including Bacteroidetes, Firmicutes, Proteobacteria, Fusobacteria, and Actinobacteria. However, the dominant bacterial genera differ between the gut and oral microbiomes. The oral cavity and gut are continuous regions connected by the GI tract. Saliva and food can also pass through the GI tract, resulting in a chemical connection between the oral and gut. Therefore, the presence of physical and chemical connections can lead to the migration of the oral microbiome toward the gut (29). Moreover, the oral microbiome might enter the circulation and colonize the GI mucosa through the hematogenous route (30). However, the oral and gut microbiomes are shown to have distinct characteristics. They might be well segregated by the presence of the oral–gut barrier, physical distance, and chemical hurdles, such as gastric acid and bile (12).

Our present study clearly showed that the observed species and Chao1 index of both gut and oral microbiomes among acute and chronic HIV-untreated groups were significantly lower than those of the control group. After 12 wk of ART, the observed species and Chao1 index of the gut microbiome increased in both acute and chronic HIV-infected groups, and no significant difference was observed between acute and chronic HIV-treated groups and control group. However, the beta diversity of the gut microbiome in acute and chronic HIV-untreated groups was significantly different from that in the control group, and the difference remained at 12 wk after the ART. Similar observations have been reported in other studies (31, 32). Altogether, these studies demonstrated that HIV infection resulted in ecological changes (relative to HIV-uninfected controls) in both alpha and beta diversity analyses, and the alpha and beta diversities were partly recovered in HIV-infected individuals after the ART. Therefore, ART might play a critical role in restoring reduced richness and diversity of the gut microbiome in HIV infection.

Most previous studies have investigated the changes of the gut microbiome in people living with chronic HIV infection. However, research on acute HIV infection is challenging due to the detection and recruitment of recently exposed individuals. Studies of the gut microbiome in HIV-untreated individuals have shown increased abundances of Proteobacteria (33 – 35) and *Prevotella* (34 – 37), while decreased abundances of Bacteroidetes (33 – 36) and Firmicutes (31, 33) were associated with chronic HIV infection. In our study, we also observed that the Proteobacteria was enriched in the chronic HIV infection, whereas in controls, the Bacteroidetes was enriched at the phylum level. In addition, the differences in gut microbiome composition between acute HIV infection and controls were also analyzed. LEfSe analyses showed that *Bacteroides fragilis*, *Sneathia*, and *Prevotella melaninogenica* were enriched in the acute HIV-infected individuals; *Porphyromonas*, *Parasutterella*, *Ruminococcus*, and *Subdoligranulum* were found in greater abundance in the control group. Notably, we found that the abundance of *Porphyromonas* in the gut microbiome was more enriched in the control group compared to the acute HIV-infected group. The results were in line with previous studies in which a lower abundance of *Porphyromonas* in the oral microbiome of HIV-infected individuals was observed in comparison with HIV-negative controls (38, 39). However, Presti et al. also showed that there is an overgrowth of *Porphyromonas* in the oral microbiome of individuals with untreated HIV (40). One possible reason for the difference might be that the study by Presti et al. was a longitudinal study without HIV-uninfected healthy controls. The surface protein of *Porphyromonas* might interact with HIV gp120, implicating the mechanism by which *Porphyromonas* prevents HIV from entering CD4^+^ T-cells (41). In addition, the binding of *Porphyromonas* to HIV might result in an easier entry of the virus into epithelial cells, facilitating oral transmission of HIV infection (42). It is known that HIV infection can lead to structural and immune disruption of the gut microbiome dysbiosis. Therefore, the decreased abundance of *Porphyromonas* in the gut microbiome might also be associated with the invasion of HIV (38). In addition, we found that acute HIV-infected individuals had lower abundance of *Ruminococcus* compared to controls, which is in line with other studies (31, 43). One study also showed that HIV infection was associated with a higher abundance of *Ruminococcus* in women with HIV infection (44). We also observed that the abundance of *Ruminococcus* in the chronic HIV-infected individuals was significantly increased compared to acute HIV-infected individuals. The abundance of *Ruminococcus* was found to be positively correlated with inflammation markers and translocation biomarker lipopolysaccharide levels (45).

Our previous study has indicated that ART can partially reverse the effects of HIV infection on the oral microbiome (25). In this longitudinal comparative study, we also evaluated the effects of ART on the gut microbiome in people with acute and chronic HIV infection. LEfSe analyses demonstrated a higher abundance of *Bacteroides* and a lower abundance of *Prevotella* in acute HIV-infected individuals after 12 wk of ART when compared with controls. These results suggested that ART initiated during acute HIV infection might play a critical role in restoring HIV-induced gut microbiota dysbiosis. Moreover, similar to changes in oral microbiome in HIV-infected individuals after ART found in our previous study (25), the abundances of potentially pathogenic bacteria in the gut microbiome of HIV-treated individuals, such as *Prevotella species*, were decreased when comparing with HIV infections before ART. However, in the chronic HIV-infected individuals after 12 wk of ART, the increased *Megamonas* and *Phascolarctobacterium* and decreased *Porphyromonas* and *Haemophilus* were observed compared with controls. Indeed, the use of ART for 12 wk might partially restore the gut microbiome during HIV infection, but it does not return the microbial composition to the normal level as that of healthy controls (46). Whether the long-time use of ART could completely restore gut microbiome composition in HIV infection remains to be shown.

Segata et al. have compared the composition and phylogenetic and metabolic characteristics of the bacterial microbiome of 10 digestive tract sites in normal adults and described the importance of the human microbiome in local and systemic diseases affecting human health (47). We also compared the composition of the oral and gut microbiome in HIV-uninfected MSM and used these data as a critical baseline to evaluate the effects of HIV and ART on the oral and gut microbiome. We found that HIV infection may cause bacterial species that do not differ between the oral and gut microbiome to become significantly different, such as *Methylobacterium–Methylorubrum*, *Megasphaera,* and *Lactobacillus*, and these differences may be able to partially recover after ART. However, ART can also impact the composition of the gut and oral microbiome, such as *Porphyromonas* and *Bradyrhizobium*. In addition, the correlation analysis revealed that the increase in the abundance of *Streptococcus* in the oral microbiome was inversely correlated with the abundance of *Streptococcus* in the gut microbiome in the HIV-infected subjects with CD4 <350 cells/µL. Our previous study has also observed increased abundance of *Streptococcus* of the oral microbiome in subjects with chronic HIV infection (25). The microbiome in the GI tract can be transmitted to the oral cavity by the fecal–oral route through direct contact and indirect exposure via contaminated objects, including water and food (48). One recent study has also shown that poor hygienic status or immunocompromised conditions might promote the fecal–oral transmission in the same individual (12). Another recent study found that *Streptococcus* was significantly enriched in the saliva of HIV-infected individuals with high plasma soluble CD14 (sCD14) levels, which might contribute to HIV-associated immune activation (49). The dysbiosis of the gut microbiome might also influence the composition of the oral microbiome and the onset of HIV-related oral and lung diseases through the effects on microbial translocation and systemic inflammation.

In this study, we found that the abundances of *Methylobacterium–Methylorubrum*, *Porphyromonas,* and *Haemophilus* collected from samples of HIV-infected subjects with CD4 <200 cells/µL were significantly lower than those in HIV-uninfected controls. Importantly, in the oral microbiome of HIV-infected individuals, we also found that lower CD4^+^ T-cell counts (<200 cells/µL) were associated with the lower abundance of *Haemophilus* (25). These findings further demonstrated that the shifts in the gut and oral microbiome might be linked to the immune status of HIV-infected individuals (40). Moreover, in the gut microbiome of PLWH, we also found that the lower CD4^+^ T-cell counts (<200 cells/µL) were associated with the higher relative abundance of *Escherichia–Shigella* and the lower relative abundance of *Methylobacterium–Methylorubrum*. *Escherichia–Shigella* belongs to *Enterobacteriaceae*. The presence of *Escherichia–Shigella* has been positively correlated with the markers, such as sCD14, IL-1β, and IFN-γ indicative of microbial translocation and systemic inflammation (50). In the SIV-infected vervet monkeys, a positive correlation between *Escherichia–Shigella* and inflammatory biomarker CXCL-9 was observed (51). Mutlu et al. found that the gut microbiome in HIV-infected individuals contained a significant increase in *Enterobacteriaceae* (52). Vujkovic-Cvijin et al. also reported that *Enterobacteriaceae* was significantly enriched in the viremic untreated HIV patients compared with HIV-uninfected controls (33). Furthermore, *Escherichia–Shigella* was negatively correlated with the CD4/CD8 ratio and positively correlated with the CD8^+^ CD57^+^ T-cells, the hallmark of immunosenescence in HIV infection (45).

The main limitation of this study was the limited sample size. One reason for the small sample size was that it was challenging to recruit newly infected acute HIV-infected individuals. The second limitation was the use of anal swab to collect fecal samples. However, several studies have shown that the stool and anal swab microbiotas from the same participant were rather similar (53 – 55). In addition, the gut and oral microbiome may be influenced by multiple factors. Studies have shown that MSM status was an important factor that might affect the HIV-related gut microbiome (56, 57). Therefore, the subjects recruited in our study were all MSM. However, our findings may also be influenced by other factors, such as age and ART regimens. Furthermore, our study was focused on the bacterial community of oral cavity and gut in HIV infection; future studies should explore the oral and gut mycobiome composition and interaction in the setting of HIV infection and ART initiation.

In conclusion, our longitudinal integrated study has shown the marked alterations in the gut and oral microbiome resulting from acute and chronic HIV infection and from ART. Importantly, the relationship between the oral and gut microbiome in people living with acute and chronic HIV infection and “healthy” controls has also been explored. These findings might contribute to a better understanding of the interactions between the oral and gut microbiome and its potential role in HIV disease progression. In addition, a deeper understanding of the composition and function of the gut and oral microbiome resulting from HIV infection and ART would help for the future development of promising biomarkers of immunodeficiency and effective microbial therapeutic strategies for HIV infection, such as probiotics, prebiotics, and synbiotics and fecal microbiota transplantation. Future studies with larger subject populations and deeper sequencing are warranted to investigate the possible mechanism of the oral and gut microbiome and their interactions affecting HIV disease progression, the reliability and sensitivity of microbial-assisted diagnosis, and the safety and efficacy of microbial-based treatment and prognosis for HIV infection.

## Data Availability

The data sets presented in this study were deposited in the NCBI Sequence Read Archive (SRA) accessible under BioProject PRJNA1013716 and the previously uploaded PRJNA739016. Other further inquiries can be directed to the corresponding author/s.

## SUPPLEMENTAL MATERIAL

Fig S1 to S4, Table S1 to S4 spectrum.01064-23-s0001.docx Comparisons of diversity, relative abundance, and pathways between gut and oral microbiome.

ASM does not own the copyrights to Supplemental Material that may be linked to, or accessed through, an article. The authors have granted ASM a non-exclusive, world-wide license to publish the Supplemental Material files. Please contact the corresponding author directly for reuse.
