# Supplementary material for: Integrated analysis of gut and oral microbiome in men who have sex with men with HIV Infection
Source: Microbiol Spectr. 2023 Oct 18;11(6):e01064-23. doi: 10.1128/spectrum.01064-23 (PMC10714972; doi:10.1128/spectrum.01064-23)
Supplement: Fig S1 to S4, Table S1 to S4 — Comparisons of diversity, relative abundance, and pathways between gut and oral microbiome. [file spectrum.01064-23-s0001.docx]

**Supplementary Materials**

| Taxonomy | Proteobacteria | Bacteroidetes | Firmicutes | Actinobacteria | Fusobacteria | Verrucomicrobiota | Cyanobacteria | Campilobacterota | Spirochaetota | Unidentified_  Bacteria | Others |
| --- | --- | --- | --- | --- | --- | --- | --- | --- | --- | --- | --- |
| Gut_Acute HIV | 0.088 | 0.506 | 0.288 | 0.057 | 0.057 | 0.000 | 0.000 | 0.002 | 0.000 | 0.001 | 0.002 |
| Gut_Chronic HIV | 0.201 | 0.432 | 0.265 | 0.028 | 0.062 | 0.000 | 0.000 | 0.004 | 0.000 | 0.001 | 0.007 |
| Gut_Non-infected | 0.081 | 0.593 | 0.249 | 0.023 | 0.038 | 0.000 | 0.001 | 0.003 | 0.000 | 0.003 | 0.010 |
| Gut_Acute ART | 0.150 | 0.415 | 0.344 | 0.032 | 0.046 | 0.000 | 0.000 | 0.002 | 0.001 | 0.002 | 0.007 |
| Gut_Chronic ART | 0.167 | 0.445 | 0.314 | 0.026 | 0.023 | 0.004 | 0.001 | 0.001 | 0.000 | 0.005 | 0.013 |
| Oral_Acute HIV | 0.280 | 0.325 | 0.327 | 0.019 | 0.045 | 0.000 | 0.000 | 0.001 | 0.001 | 0.000 | 0.001 |
| Oral_Chronic HIV | 0.308 | 0.283 | 0.359 | 0.011 | 0.035 | 0.000 | 0.000 | 0.002 | 0.001 | 0.001 | 0.001 |
| Oral_Non-infected | 0.313 | 0.211 | 0.354 | 0.059 | 0.038 | 0.000 | 0.005 | 0.001 | 0.008 | 0.005 | 0.005 |
| Oral_Acute ART | 0.408 | 0.201 | 0.345 | 0.014 | 0.029 | 0.000 | 0.000 | 0.001 | 0.001 | 0.000 | 0.001 |
| Oral_Chronic ART | 0.387 | 0.232 | 0.310 | 0.021 | 0.039 | 0.000 | 0.002 | 0.002 | 0.003 | 0.002 | 0.001 |

**Table S1.** Relative abundance of oral and gut microbiome in HIV-infected groups and controls at the phylum level. Oral_Acute HIV: oral microbiome in people living with acute HIV infection at baseline; Oral_Chronic HIV: oral microbiome in people living with chronic HIV infection at baseline; Oral_Non-infected: oral microbiome in HIV-uninfected controls; Oral_Acute ART: oral microbiome in people living with acute HIV infection after 12 weeks of ART; Oral_Chronic ART: oral microbiome in people living with chronic HIV infection after 12 weeks of ART. Gut_Acute HIV: gut microbiome in people living with acute HIV infection at baseline; Gut_Chronic HIV: gut microbiome in people living with chronic HIV infection at baseline; Gut_Non-infected: gut microbiome in HIV-uninfected controls; Gut_Acute ART: gut microbiome in people living with acute HIV infection after 12 weeks of ART; Gut_Chronic ART: gut microbiome in people living with chronic HIV infection after 12 weeks of ART.

| Taxonomy | *Escherichia*  *-Shigella* | *Prevotella* | *Bacteroides* | *Bradyrhizobium* | *Streptococcus* | *Neisseria* | *Methylobacterium*  *-Methylorubrum* | *Actinobacillus* | *Klebsiella* | *Haemophilus* | Others |
| --- | --- | --- | --- | --- | --- | --- | --- | --- | --- | --- | --- |
| Gut_Acute HIV | 0.058 | 0.434 | 0.049 | 0.000 | 0.060 | 0.000 | 0.004 | 0.000 | 0.006 | 0.002 | 0.387 |
| Gut_Chronic HIV | 0.148 | 0.386 | 0.023 | 0.000 | 0.014 | 0.001 | 0.009 | 0.000 | 0.006 | 0.007 | 0.407 |
| Gut_Non-infected | 0.030 | 0.495 | 0.044 | 0.000 | 0.022 | 0.000 | 0.027 | 0.000 | 0.003 | 0.001 | 0.378 |
| Gut_Acute ART | 0.101 | 0.285 | 0.099 | 0.000 | 0.071 | 0.000 | 0.021 | 0.000 | 0.006 | 0.001 | 0.417 |
| Gut_Chronic ART | 0.064 | 0.385 | 0.038 | 0.000 | 0.018 | 0.000 | 0.039 | 0.000 | 0.026 | 0.000 | 0.430 |
| Oral_Acute HIV | 0.000 | 0.240 | 0.000 | 0.000 | 0.196 | 0.175 | 0.001 | 0.010 | 0.000 | 0.074 | 0.304 |
| Oral_Chronic HIV | 0.000 | 0.223 | 0.000 | 0.000 | 0.249 | 0.179 | 0.000 | 0.042 | 0.000 | 0.079 | 0.228 |
| Oral_Non-infected | 0.000 | 0.126 | 0.003 | 0.000 | 0.161 | 0.187 | 0.001 | 0.032 | 0.000 | 0.058 | 0.432 |
| Oral_Acute ART | 0.000 | 0.105 | 0.000 | 0.043 | 0.258 | 0.205 | 0.000 | 0.024 | 0.000 | 0.121 | 0.244 |
| Oral_Chronic ART | 0.000 | 0.145 | 0.000 | 0.068 | 0.220 | 0.182 | 0.000 | 0.022 | 0.000 | 0.087 | 0.276 |

**Table S2.** Relative abundance of oral and gut microbiome in HIV-infected groups and controls at the genus level. Oral_Acute HIV: oral microbiome in people living with acute HIV infection at baseline; Oral_Chronic HIV: oral microbiome in people living with chronic HIV infection at baseline; Oral_Non-infected: oral microbiome in HIV-uninfected controls; Oral_Acute ART: oral microbiome in people living with acute HIV infection after 12 weeks of ART; Oral_Chronic ART: oral microbiome in people living with chronic HIV infection after 12 weeks of ART. Gut_Acute HIV: gut microbiome in people living with acute HIV infection at baseline; Gut_Chronic HIV: gut microbiome in people living with chronic HIV infection at baseline; Gut_Non-infected: gut microbiome in HIV-uninfected controls; Gut_Acute ART: gut microbiome in people living with acute HIV infection after 12 weeks of ART; Gut_Chronic ART: gut microbiome in people living with chronic HIV infection after 12 weeks of ART.

| **Group** | **Observed species** | **Shannon** | **Simpson** | **Chao1** | **ACE** | **Goods coverage** | **PD whole**  **tree** |
| --- | --- | --- | --- | --- | --- | --- | --- |
| Gut_Acute HIV | 412 | 4.504 | 0.860 | 507.057 | 508.429 | 0.996 | 33.329 |
| Gut_Chronic HIV | 421 | 4.526 | 0.835 | 512.926 | 516.370 | 0.996 | 34.373 |
| Gut_Non-infected | 470 | 4.675 | 0.841 | 584.216 | 598.791 | 0.995 | 39.928 |
| Gut_Acute ART | 492 | 4.522 | 0.826 | 622.515 | 636.211 | 0.995 | 45.036 |
| Gut_Chronic ART | 512 | 4.708 | 0.863 | 643.685 | 658.073 | 0.995 | 47.748 |
| Oral_Acute HIV | 258 | 4.347 | 0.879 | 318.163 | 319.952 | 0.998 | 22.127 |
| Oral_Chronic HIV | 236 | 4.169 | 0.868 | 283.989 | 285.901 | 0.998 | 19.425 |
| Oral_Non-infected | 579 | 5.794 | 0.943 | 675.910 | 688.982 | 0.995 | 44.576 |
| Oral_Acute ART | 263 | 4.146 | 0.860 | 311.125 | 312.678 | 0.998 | 23.302 |
| Oral_Chronic ART | 271 | 4.095 | 0.848 | 321.480 | 324.644 | 0.998 | 25.201 |

**Table S3.** The Observed-species, Chao1, Shannon, Simpson, ACE, Good-coverage and PD whole tree indices were used to estimate Alpha diversity. Oral_Acute HIV: oral microbiome in people living with acute HIV infection at baseline; Oral_Chronic HIV: oral microbiome in people living with chronic HIV infection at baseline; Oral_Non-infected: oral microbiome in HIV-uninfected controls; Oral_Acute ART: oral microbiome in people living with acute HIV infection after 12 weeks of ART; Oral_Chronic ART: oral microbiome in people living with chronic HIV infection after 12 weeks of ART. Gut_Acute HIV: gut microbiome in people living with acute HIV infection at baseline; Gut_Chronic HIV: gut microbiome in people living with chronic HIV infection at baseline; Gut_Non-infected: gut microbiome in HIV-uninfected controls; Gut_Acute ART: gut microbiome in people living with acute HIV infection after 12 weeks of ART; Gut_Chronic ART: gut microbiome in people living with chronic HIV infection after 12 weeks of ART.

| **Group** | **Observed species**  **(*p* values)** | **Chao1**  **(*p* values)** | **Unweighted UniFrac (*p* values)** | **Weighted UniFrac**  **(*p* values)** |
| --- | --- | --- | --- | --- |
| **Gut_Acute HIV** vs **Gut_Non-infected** | 0.0054 | 0.0169 | 0.0041 | 0.0031 |
| **Gut_Chronic HIV** vs **Gut_Non-infected** | 0.0271 | 0.0239 | 0.7658 | <0.0001 |
| **Gut_Acute HIV** vs **Gut_Chronic HIV** | 0.5541 | 0.8933 | 0.0016 | 0.5935 |
| **Gut_Acute ART** vs **Gut_Non-infected** | 0.7266 | 0.661 | <0.0001 | <0.0001 |
| **Gut_Chronic ART** vs **Gut_Non-infected** | 0.3522 | 0.1152 | <0.0001 | <0.0001 |
| **Gut_Acute HIV** vs **Gut_Acute ART** | 0.019 | 0.0064 | <0.0001 | 0.0054 |
| **Gut_Chronic HIV** vs **Gut_Chronic ART** | 0.0019 | <0.0001 | <0.0001 | 0.1959 |
| **Gut_Acute ART** vs **Gut_Chronic ART** | 0.2135 | 0.2784 | 0.0528 | 0.2722 |

**Table S4.** Comparisons of the alpha diversity (Observed species and Chao1) and beta diversity (Unweighted UniFrac and Weighted UniFrac) in gut and oral microbiome among groups. The Wilcoxon rank-sum test was used and *p*<0.05 was considered to be statistically significant. Oral_Acute HIV: oral microbiome in people living with acute HIV infection at baseline; Oral_Chronic HIV: oral microbiome in people living with chronic HIV infection at baseline; Oral_Non-infected: oral microbiome in HIV-uninfected controls; Oral_Acute ART: oral microbiome in people living with acute HIV infection after 12 weeks of ART; Oral_Chronic ART: oral microbiome in people living with chronic HIV infection after 12 weeks of ART. Gut_Acute HIV: gut microbiome in people living with acute HIV infection at baseline; Gut_Chronic HIV: gut microbiome in people living with chronic HIV infection at baseline; Gut_Non-infected: gut microbiome in HIV-uninfected controls; Gut_Acute ART: gut microbiome in people living with acute HIV infection after 12 weeks of ART; Gut_Chronic ART: gut microbiome in people living with chronic HIV infection after 12 weeks of ART.


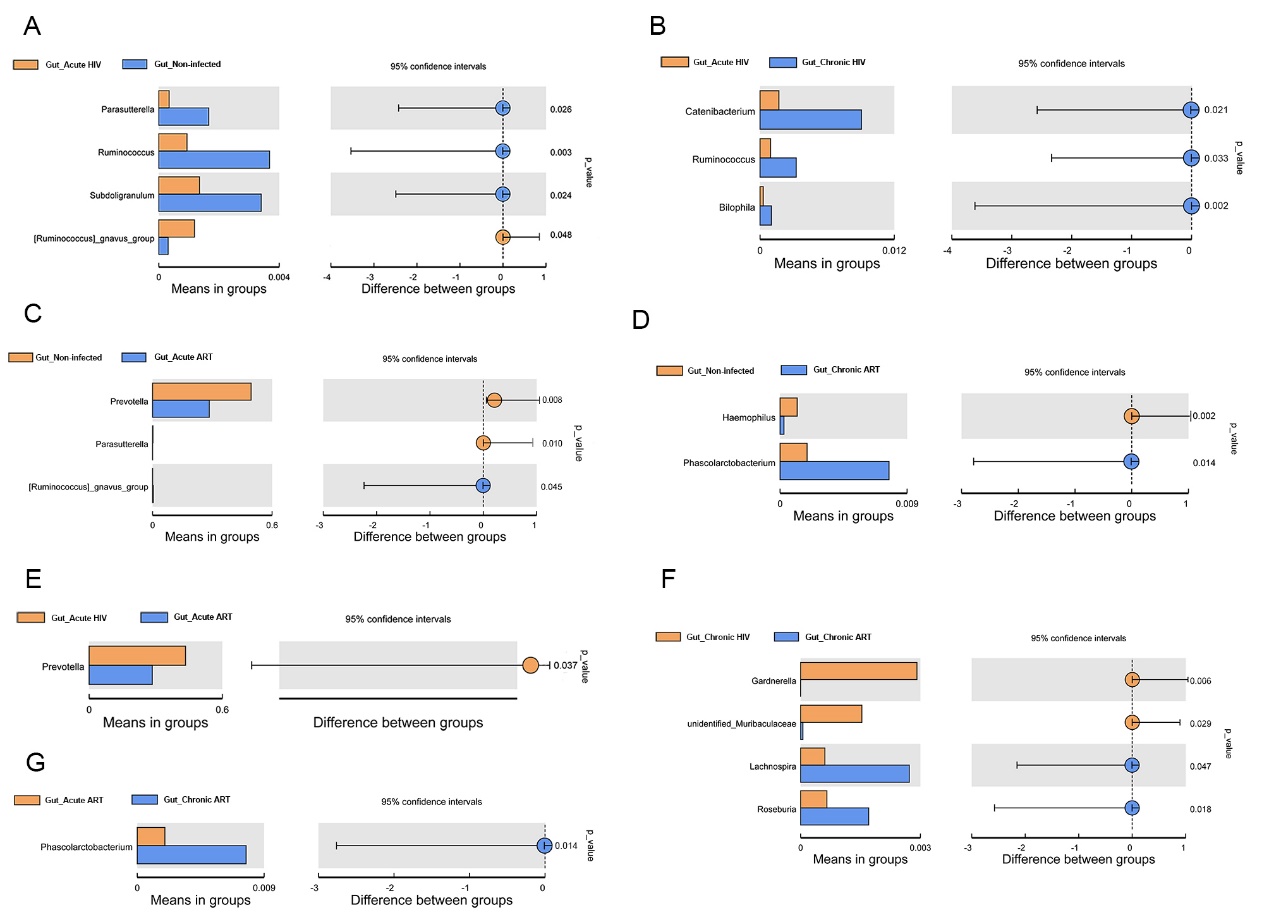


**Figure S1.** Comparisons of the relative abundance in gut microbiome at the genus level (A) Between Gut_Acute HIV and Gut_Non-infected groups; (B) Between Gut_Acute HIV and Gut_Chronic HIV groups; (C) Between Gut_Acute ART and Gut_Non-infected groups; (D) Between Gut_Chronic ART and Gut_Non-infected groups; (E) Between Gut_Acute HIV and Gut_Acute ART groups; (F) Between Gut_Chronic HIV and Gut_Chronic ART groups; (G) Between Gut_Acute ART and Gut_Chronic ART groups. The T-test was used and *p*<0.05 was considered to be statistically significant.


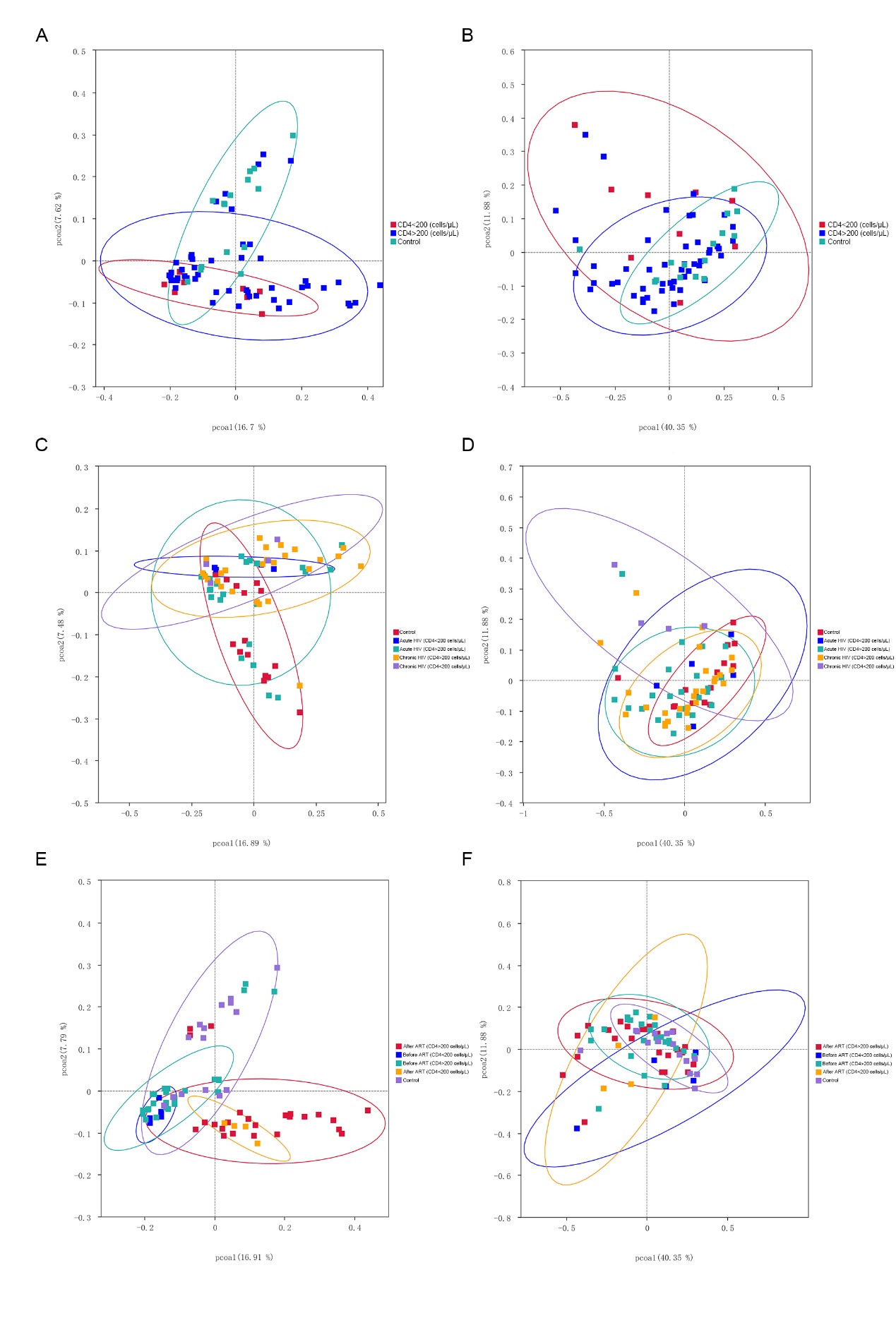


**Figure S2.** The diversity of gut microbiome in HIV-infected subjects with CD4<200 cells/μL and CD4>200 cells/μL groups and controls. (A) Beta diversity represented by Principal coordinate analysis (PcoA) of unweighted UniFrac distances between HIV-infected groups (HIV-infected subjects with CD4<200 cells/μL and CD4>200 cells/μL) and controls; (B) Beta diversity represented by PcoA of weighted UniFrac distances between HIV-infected groups (HIV-infected subjects with CD4<200 cells/μL and CD4>200 cells/μL) and controls. (C) Beta diversity represented by PcoA of unweighted UniFrac distances between acute and chronic HIV-infected groups (HIV-infected subjects with CD4<200 cells/μL and CD4>200 cells/μL) and controls; (D) Beta diversity represented by PcoA of weighted UniFrac distances between acute and chronic HIV-infected groups (HIV-infected subjects with CD4<200 cells/μL and CD4>200 cells/μL) and controls. (E) Beta diversity represented by PcoA of unweighted UniFrac distances between HIV-infected before and after ART groups (HIV-infected subjects with CD4<200 cells/μL and CD4>200 cells/μL) and controls; (F) Beta diversity represented by PcoA of weighted UniFrac distances HIV-infected before and after ART groups (HIV-infected subjects with CD4<200 cells/μL and CD4>200 cells/μL) and controls.

**
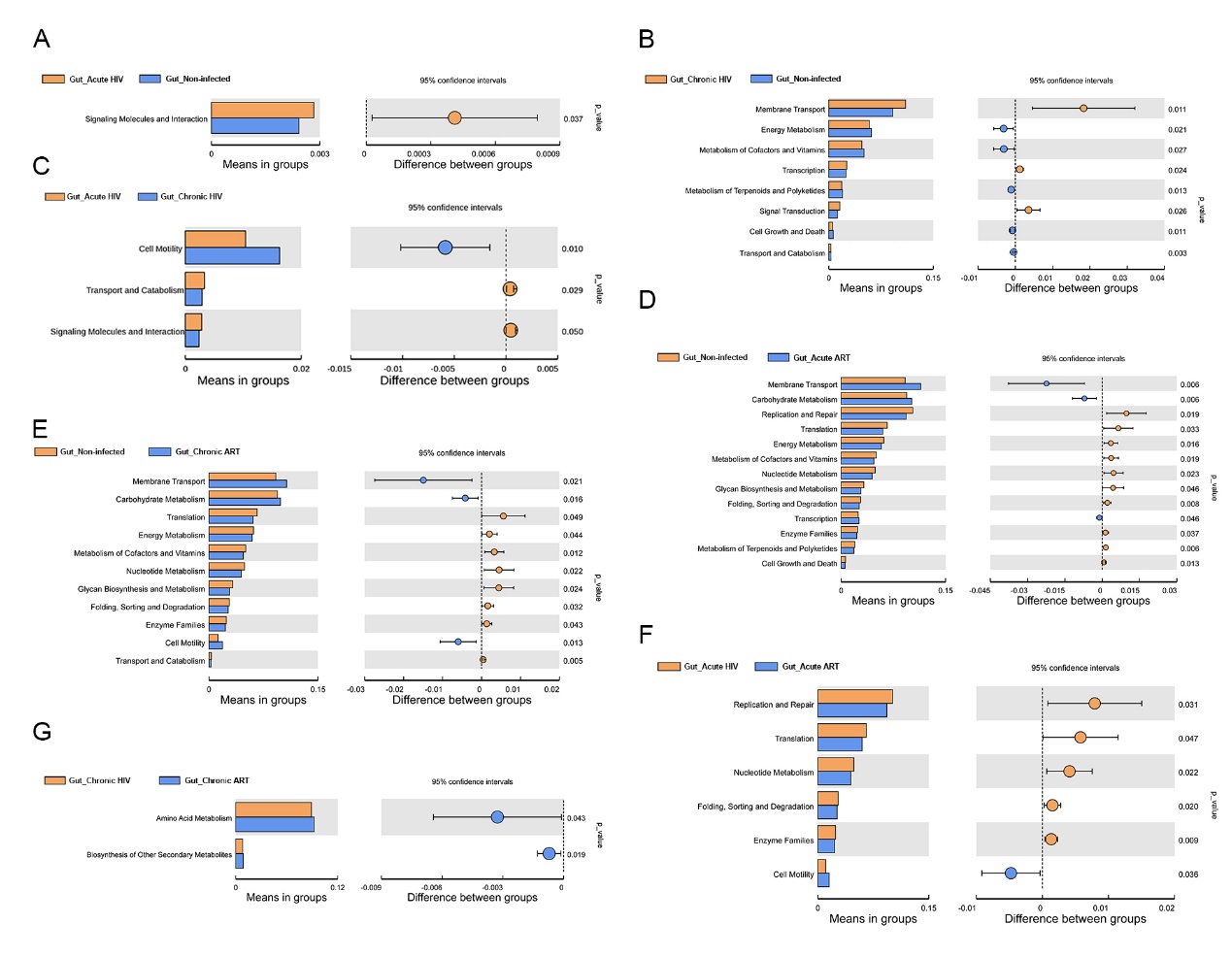
**

**Figure S3.** Comparisons of the pathways (A) between Gut_Acute HIV and Gut_Non-infected groups; (B) between Gut_Chronic HIV and Gut_Non-infected groups; (C) between Gut_Acute HIV and Gut_Chronic HIV groups； (D) between Gut_Acute ART and Gut_Non-infected groups; (E) between Gut_Chronic ART and Gut_Non-infected groups; (F) between Gut_Acute HIV and Gut_Acute ART groups; (G) between Gut_Chronic HIV and Gut_Chronic ART groups. The T-test was used and *p*<0.05 was considered to be statistically significant.


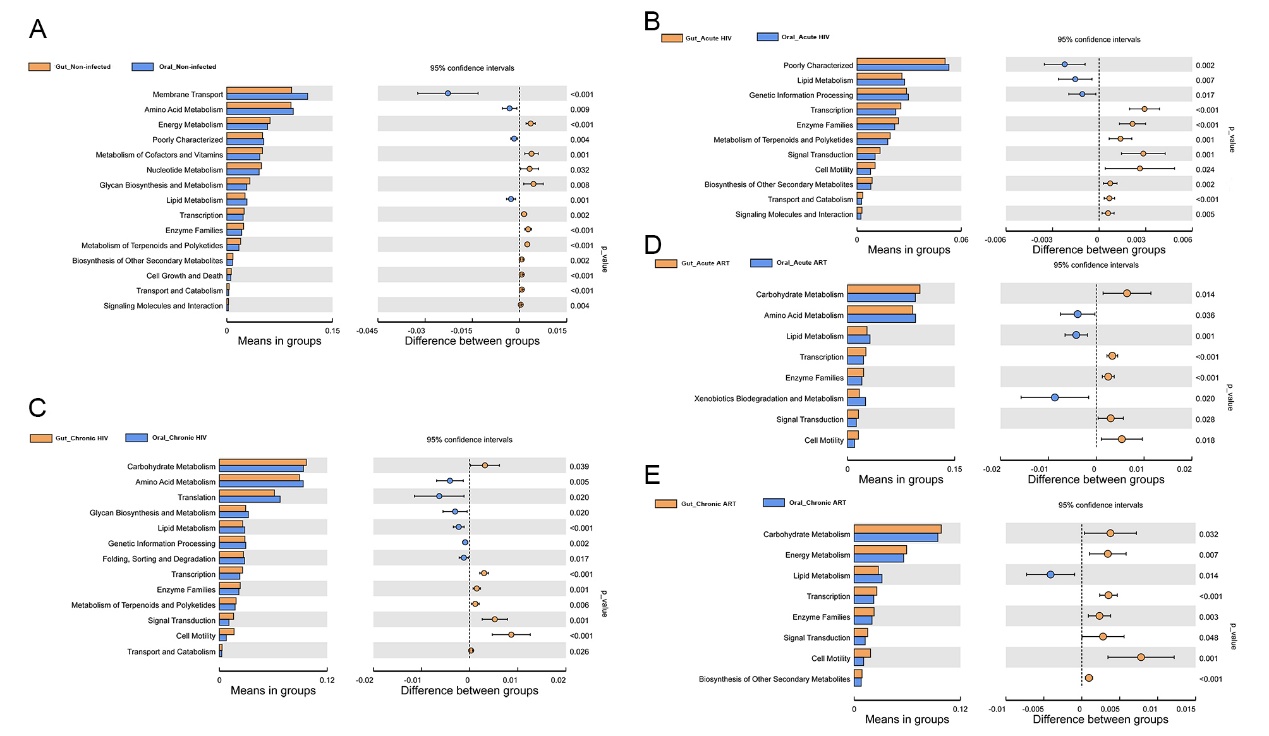


**Figure S4.** Comparisons of the pathways (A) between Oral_Non-infected and Gut_Non-infected groups; (B) between Gut_Acute HIV and Oral_Acute HIV groups; (C) between Gut_Chronic HIV and Oral_Chronic HIV groups; (D) between Gut_Acute ART and Oral_Acute ART groups; (E) between Gut_Chronic ART and Oral_Chronic ART groups. The T-test was used and *p*<0.05 was considered to be statistically significant.
